# Flexible cellulose and ZnO hybrid nanocomposite and its UV sensing characteristics

**DOI:** 10.1080/14686996.2017.1336642

**Published:** 2017-06-26

**Authors:** Seongcheol Mun, Hyun Chan Kim, Hyun-U Ko, Lindong Zhai, Jung Woong Kim, Jaehwan Kim

**Affiliations:** ^a^ Creative Research Center for Nanocellulose Future Composites, Department of Mechanical Engineering, Inha University, Incheon, Republic of Korea

**Keywords:** Hybrid nanocomposite, cellulose, ZnO nanorod, organic-inorganic composite, UV sensing, flexible sensor, 20 Organic and soft materials (colloids, liquid crystals, gel, polymers), 103 Composites, 102 Porous / Nanoporous / Nanostructured materials, 204 Optics / Optical applications, 208 Sensors and actuators

## Abstract

This paper reports the synthesis and UV sensing characteristics of a cellulose and ZnO hybrid nanocomposite (CEZOHN) prepared by exploiting the synergetic effects of ZnO functionality and the renewability of cellulose. Vertically aligned ZnO nanorods were grown well on a flexible cellulose film by direct ZnO seeding and hydrothermal growing processes. The ZnO nanorods have the wurtzite structure and an aspect ratio of 9 ~ 11. Photoresponse of the prepared CEZOHN was evaluated by measuring photocurrent under UV illumination. CEZOHN shows bi-directional, linear and fast photoresponse as a function of UV intensity. Electrode materials, light sources, repeatability, durability and flexibility of the prepared CEZOHN were tested and the photocurrent generation mechanism is discussed. The silver nanowire coating used for electrodes on CEZOHN is compatible with a transparent UV sensor. The prepared CEZOHN is flexible, transparent and biocompatible, and hence can be used for flexible and wearable UV sensors.

## Graphical abstract

**Figure:**
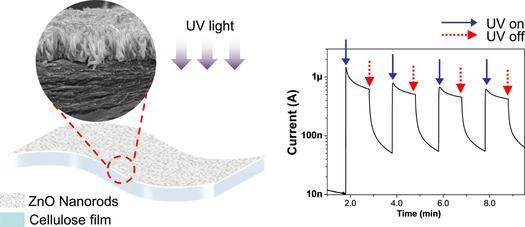

## 1. Introduction

Nowadays, environmentally friendly materials have gained much attention because of pollution, a shortage of resources and industrial waste. Cellulose, a natural polymer material, is a strong candidate for environmentally friendly composite materials because of its merits in terms of its eco-friendly, abundant, biodegradable, renewable and biocompatible characteristics [1,2]. Cellulose has been discovered to be a smart material, namely electro-active paper (EAPap) and various applications of EAPap have been reported, such as energy harvesters, sensors and actuators. EAPap has versatile properties which are useful for a wide range of applications because of its light weight, transparency, flexibility, piezoelectricity, high mechanical strength and biodegradability [2–5]. In order to enhance performance and confer new functionality of EAPap, inorganic nanomaterials have been employed in cellulose composite films. Titanium oxide (TiO_2_), tin oxide (SnO), iron oxide (Fe_2_O_3_), graphene oxide (GO) and carbon nanotube (CNT) were blended/bonded with cellulose to comprise cellulose-based multifunctional composite materials [6–10]. Such multifunctional composite materials enhanced their mechanical and chemical properties, which expanded their applications to gas sensors, biosensors, mechanical reinforced films, energy materials and flexible field effect transistors.

Zinc oxide (ZnO) is an attractive functional nanomaterial with a wide band gap (E_g_ = 3.4 eV), large free exciton energy (60 meV), piezoelectricity, photoelectrical property and stability. ZnO is an important nanomaterial in electronic and photonic devices. Additionally, ZnO is useful for bio-electronics and the cosmetic industry due to its biocompatibility and environmental friendliness [11–13]. ZnO can be formed as various shapes such as nanorod, nanowire, nanobelt, nanoneedle, nanotube and nanoplate. Depending on the process conditions, they reveal different properties which lead to a wide range of applications [14–19]. Among them, well-grown nanorods are the most promising structure since it shows superior characteristics to others. Many fabrication methods in ZnO grown nanorods are available such as chemical vapor deposition, metal organic chemical vapor deposition, pulsed laser deposition, vapor liquid solid epitaxial, physical vapor deposition and spray pyrolysis [20–24]. Although these methods are well developed and result in a good nanoscale structure, they are complex due to requiring high vacuum, high temperatures and expensive equipment. The aqueous solution-based hydrothermal process has received attention due to the simplicity of the process, low temperature, large-scale fabrication and low cost [25–28].

Recently, nanomaterials have been studied to improve functionality and the properties of polymers, namely organic-inorganic hybrid composites which exhibit the combined synergistic effect of inorganic nanomaterials and organic polymers. With increasing interest in wearable electronics, the demand of organic-inorganic hybrid composites is thus increased. ZnO is an attractive nanomaterial for the hybrid composites because it can be easily grown on various substrates including metal, glass, silicon, sapphire, flexible plastic and polymers [19,29–32]. ZnO-polymer hybrid composites have been developed to bridge the semiconducting properties of ZnO and flexibility of the polymer [26,28–31]. A flexible ZnO energy harvester and nanogenerator can generate electrical energy by large deformation under stretching or compression load due to the substrate flexibility [33,34]. A flexible ZnO strain sensor can be utilized to structure a health monitoring system of arbitrarily shaped structures [35,36]. Polymer-based ZnO thin film transistors, surface acoustic wave devices and dye-sensitized solar cells can be realized by applying flexible electronics [37–39]. ZnO-polymer hybrid composites have been investigated as gas, humidity and UV sensors for wearable electronics [31,40–42]. However, in-depth investigation of UV sensing behavior of flexible, transparent and renewable cellulose-ZnO hybrid nanocomposite (CEZOHN) has not been performed. Furthermore, the UV sensing behavior of ZnO nanorods grown on a cellulose film has not been investigated.

This paper explores fabricating a flexible, transparent and renewable cellulose-ZnO hybrid nanocomposite (CEZOHN) and investigating the UV photoresponse of the prepared CEZOHN in terms of electrode materials, light sources, repeatability, durability and flexibility. Two-step fabrication processes of ZnO nanorods are utilized by direct ZnO seeding and hydrothermal ZnO nanorod growth on the cellulose film. The morphology and structure of grown ZnO nanorods are analyzed by scanning electron microscopy (SEM), X-ray diffraction (XRD), high resolution transmission electron microscopy (HRTEM), atomic force microscopy (AFM), and Fourier transform infrared (FTIR) spectroscopy. Optical properties are investigated by measuring photoluminescence (PL) and UV-visible absorption spectra. The UV photoresponse of the CEZOHN is investigated by taking into account the exposure side, light intensity, light source and electrode materials. Repeatability, durability and flexibility of the CEZOHN are investigated and the photocurrent generation mechanism is explained.

## 2. Experimental details

### 2.1. Fabrication

Cellulose ZnO nanorod hybrid nanocomposite was prepared in two steps: ZnO seeding and ZnO nanorod growth on a cellulose film. The detailed fabrication process of the cellulose film is reported in the references [2,3]. Briefly, dried cotton pulp (degree of polymerization 4500, Buckeye Technologies Inc., Memphis, TN, USA) was torn into small pieces and then mixed with LiCl in N, N-dimethylacetamide (DMAc, Sigma-Aldrich, St. Louis, MO, USA) for dissolving cellulose fiber. A transparent cellulose solution was obtained by heating up to 150 °C while stirring mechanically according to the solvent exchange. The cellulose solution was poured on a glass plate and cast by a doctor blade. A wet cellulose film was obtained after curing by using deionized (DI) water/isopropyl alcohol (IPA) mixture followed by DI water washing for one day. The wet cellulose film was dried on Styrofoam with clamping to maintain its flatness in an ambient condition. The dried cellulose film was fixed on a silicon wafer with Kapton tape. A solvent for the ZnO seeding was prepared by dissolving 50 mM of zinc acetate dihydrate (Zn(CH_3_COO)_2_·2H_2_O, Sigma-Aldrich, St. Louis, MO, USA) in ethyl alcohol. The first ZnO seed layer was formed on the surface of the cellulose film by spin coating the solvent, followed by drying at 100 °C. The spin coating was repeated 10 times every 3 minutes to provide a sufficient ZnO seed layer, and then 30 minutes of extra heating was supplied to remove any remnant solvent and residues. For the ZnO growth, another ZnO-derived solvent was prepared by mixing 50 mM of zinc nitrate hexahydrate (Zn(NO_3_)_2_·6H_2_O, Sigma-Aldrich, St. Louis, MO, USA) and hexamethylenetetramine (HMT, (CH_2_)_6_·N_4_, Sigma-Aldrich, St. Louis, MO, USA), both dissolved in DI water. When the ZnO seeded cellulose was immersed into the derived solvent at 90 °C for 1 hour, ZnO nanoparticles were grown on ZnO nanorods. After the growth, the film was rinsed and dried on the Styrofoam at room temperature for one day, which resulted in CEZOHN.

### 2.2. Characterization

The sample morphology was monitored using a field-emission SEM (S-4300, Hitachi, Tokyo, Japan). An AFM (Dimension-3000, Veeco, Plainview, NY, USA) was employed to analyze the surface morphology and size of the seeds and ZnO nanorods in tapping mode. To investigate the crystal structure of ZnO nanorods, XRD patterns were acquired from thin films using a DMAX-2500 spectrometer (Rigaku, Tokyo, Japan), Cu-Ka radiation, 2θ angle mode and 0.02° step. Optical properties were characterized via UV-visible (8452A, HP, Palo Alto, CA, USA, 190–800 nm range) and FTIR absorption (FTS-3000, Bio-Rad, Hercules, CA, USA). The crystalline structure and morphology were also studied by HRTEM (3010, JEOL, Tokyo, Japan). The PL of CEZOHN was measured at - 50 °C using a Maple-I (Dongwoo Optron, Gwangju-si, Gyeonggi-do, South Korea) with a 325 nm He-Cd laser.

### 2.3. UV photoresponse test

To evaluate UV photoresponse, platinum electrodes were deposited on both sides of the prepared CEZOHN by using a magnetron sputter (K-575X, EMI Tech, Lewes, England). The electrode size is 1 cm × 4 cm, and a wire was connected to each electrode. The prepared CEZOHN was attached to a flexible and transparent plate and UV light was exposed to the cellulose side or ZnO side of CEZOHN. The UV light lamp (PL-S 9 W/2P BLB, Philips, Amsterdam, Netherlands) was fixed with a standing jig and the intensity of UV light exposure was controlled by the distance to the CEZOHN. The source of the UV lamp was 365 nm wavelength and the power density was measured by using a UV meter (UV-A Meter, Kuhnast, Wachterbach, Germany) which has the highest sensitivity at 360 nm. The CEZOHN was connected to the picoammeter and a pulse analyzer was used to monitor the induced current during the UV light exposure.

## 3. Results and discussion

### 3.1. Characterization

**Figure 1.**
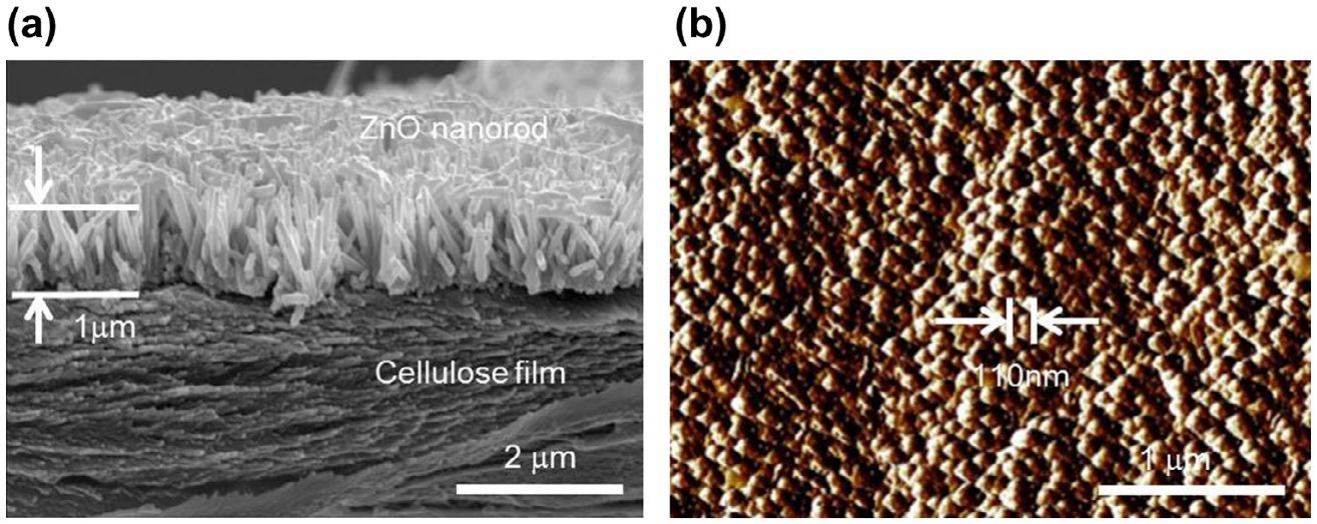
Morphology investigation of the prepared CEZOHN: (a) cross-sectional SEM image and (b) AFM surface image**.**

Morphologies of the prepared CEZOHN were investigated by taking SEM. Figure 1(a) shows the cross-sectional SEM image of the CEZOHN. High-density ZnO nanorods are shown on the cellulose film vertically and uniformly grown. From the SEM image and the AFM image shown in Figure 1(b), ZnO nanorods are found to be 1 μm long with a 110 nm diameter. The aspect ratio of ZnO nanorods ranges from 9 to 11. The high aspect ratio of ZnO nanorods is beneficial for electrical properties as it results in the fast movement of charge carriers [43].

**Figure 2.**
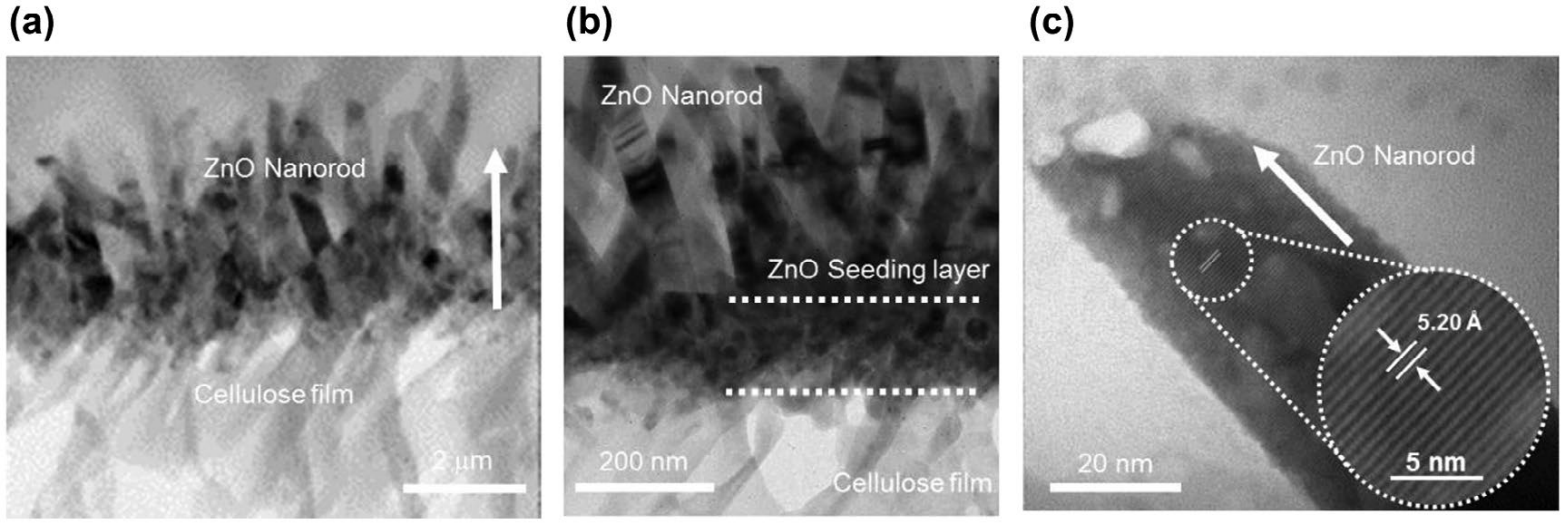
TEM images of (a) the CEZOHN with (b) interface morphology and (c) a high resolution image of a ZnO nanorod.

Detailed structural characteristics of the CEZOHN and ZnO nanorods were evaluated by HRTEM. Figure 2(a) shows the interface boundary between the cellulose film and the ZnO nanorod. ZnO nanorods were grown to the vertical direction on the surface of the cellulose film. Figure 2(b) shows the TEM image of the ZnO seeding layer on the cellulose film. The seeding layer was formed at the bottom of the ZnO nanorods with a 150 nm thickness. Figure 2(c) shows a high resolution TEM image of a single ZnO nanorod. From the image the lattice space of ZnO was estimated at 5.2 Å, which corresponds to the (002) planes in the wurtzite structure. The wurtzite structure of ZnO is beneficial to the electrical properties of CEZOHN.

**Figure 3.**
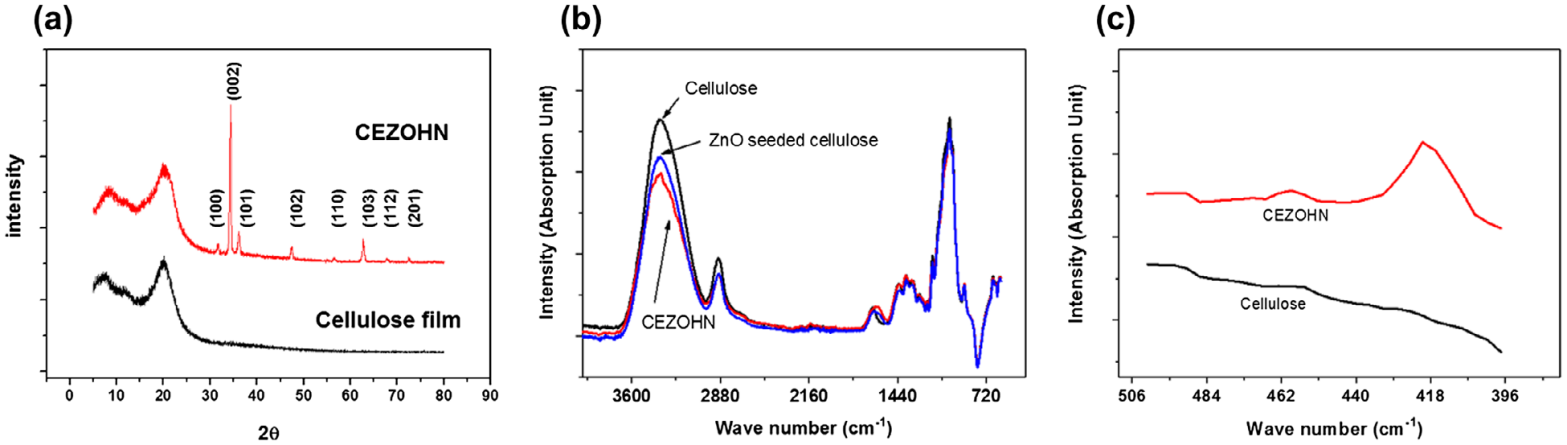
(a) XRD patterns and (b), (c) FTIR absorption spectra of CEZOHN and cellulose film.

In order to further investigate the crystal structure of CEZOHN, XRD measurements were carried out. Figure 3(a) shows the XRD curves of the CEZOHN. The broad XRD peak around 21° represents a typical characteristic peak of regenerated cellulose, while several diffraction peaks at 31.7°, 34.4° and 36.2° indicate (100), (002), (101) wurtzite crystalline structures of the ZnO nanorod. The highest peak detected at 34.4° indicates that most ZnO nanorods are grown vertically on the cellulose film as followed to (002) plane. To investigate the chemical function groups, FTIR was taken. Figure 3(b) shows the comparison of FTIR curves between the cellulose film and the CEZOHN. A significant difference is detected at 3370 cm^−1^, which is assigned to the peak of the hydroxyl group. The prepared CEZOHN shows remarkably low peak intensity at 3370 cm^−1^ compared to the cellulose film, suggesting that the hydroxyl groups were mostly consumed by a reaction with zinc ion and water molecules during the seeding and growing processes. The peak of chemical bonding with Zn-O is also observed at 420 cm^−1^, which was not detected from the cellulose film in Figure 3(c) [44].

**Figure 4.**
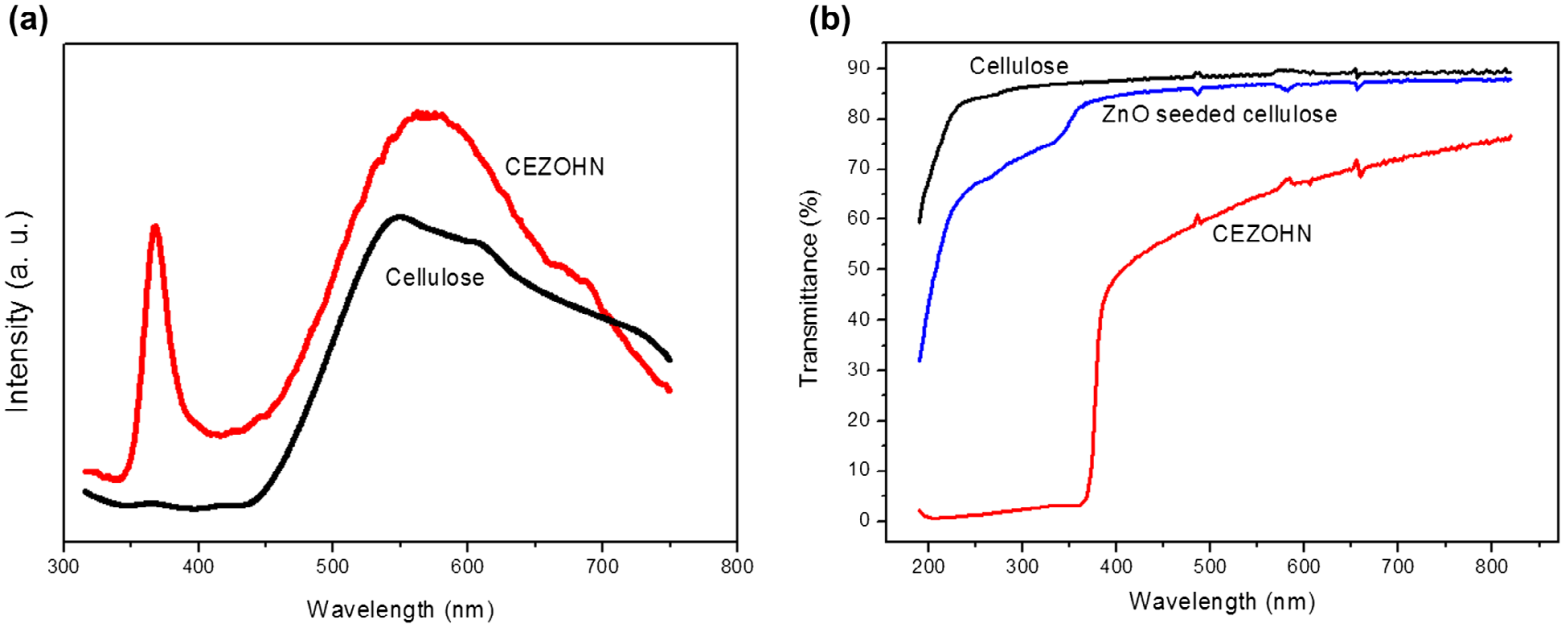
(a) PL curves and (b) UV-visible transmittance spectra of cellulose film and CEZOHN.

The PL property of the cellulose film and the CEZOHN was taken. Figure 4(a) shows the PL curves of the cellulose film and CEZOHN. Cellulose film has a broad PL peak at around 550 nm. The PL spectra of CEZOHN show two peaks. The broad peak is detected from 450 to 750 nm, which is deep-level-emission (DLE) range and mainly associated with oxygen vacancies of ZnO [31]. This broad peak is due to the formation of crystal defects made by the low temperature fabrication process. The sharp luminescence peak detected at 373 nm corresponds to the near-band-edge emission (NBE) of ZnO [27,31,45–47]. Note that the PL curve of the CEZOHN is not much influenced by the cellulose film.

Optical transparency was analyzed by measuring UV-visible spectra of the cellulose film, the ZnO seeded cellulose and the CEZOHN. Figure 4(b) shows UV-visible spectra in the wavelength between 200 and 800 nm. The UV-visible spectrum of the cellulose film shows good transparency over 85%. Note that the ZnO seeded cellulose film shows transparency less than 85% compared to the cellulose film in visible light range. Below 380 nm, more absorption can be seen from the CEZOHN, which is attributed to the ZnO seed layer. Although most UV light is absorbed by ZnO nanorods, the optical property of the CEZOHN is acceptable in comparison with other organic-inorganic hybrid composites [48–50].

### 3.2. UV photoresponse

#### 3.2.1. UV intensity effect

**Figure 5.**
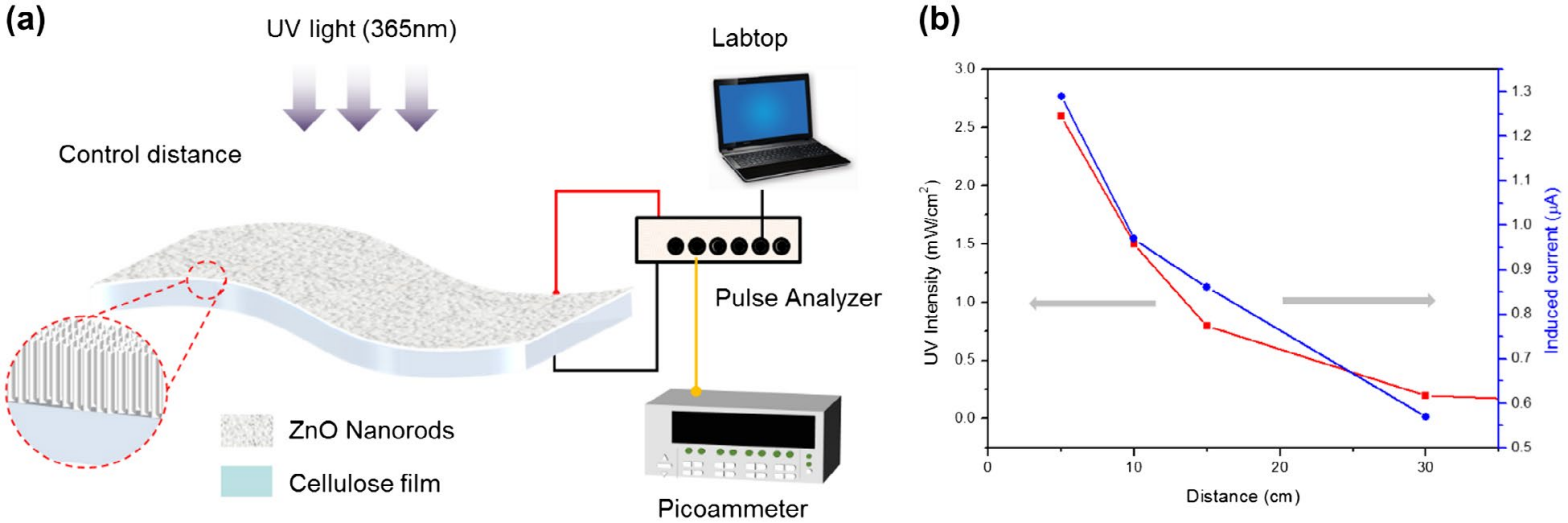
(a) Schematic of UV photoresponse test setup (b) UV intensity at various distances from the UV source**.**

**Figure 6.**
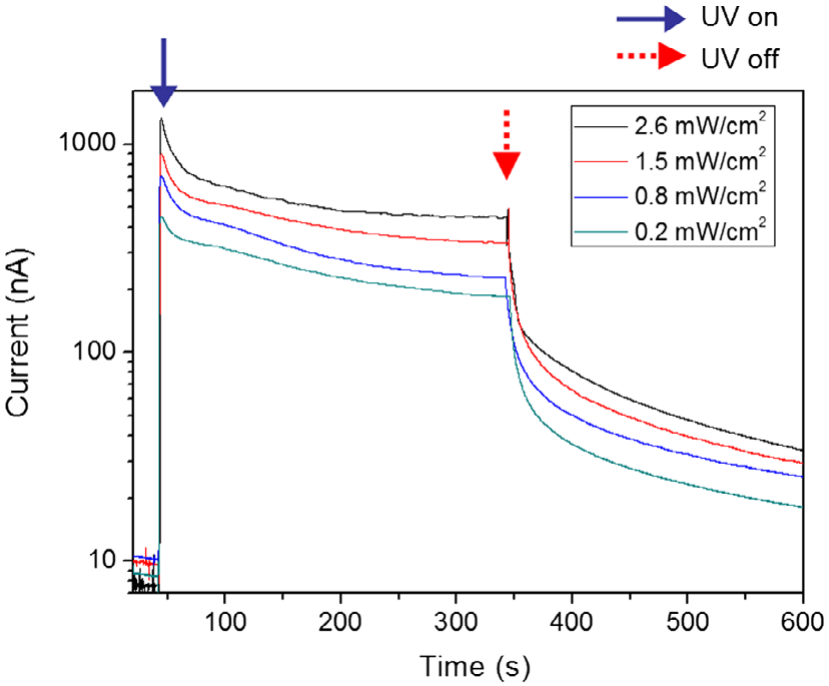
Photocurrent outputs at various UV intensities.

The prepared CEZOHN requires conductive electrodes to measure UV-induced electrical response. Various metals such as Al, Au and Pt can be candidates for the electrode deposition and Pt was chosen at first. Figure 5 shows the schematic of the UV photoresponse setup and UV light intensity as a function of distance from the CEZOHN. The UV intensity is inversely proportional to the distance from the CEZOHN. The intensity is inversely proportional to the distance, and it was varied between 0.2 and 2.6 mW/cm^2^ by changing the distance in the photoresponse experiments. The induced current increased as UV intensity as shown in Figure 6. As a function of time, it spiked after exposure to light and then gradually decreased. The peak current values were 1.34, 0.90, 0.70 and 0.40 μA at the power densities of 2.6, 1.5, 0.8 and 0.2 mW/cm^2^, respectively. Carrier generation in ZnO by UV light is generally explained by a trapping mechanism based on the absorption and desorption of oxygen molecules at the surface [29]. The decrease in current with UV exposure time might be due to a recombination between photon-generated holes and reabsorbed oxygen molecules; this decrease saturates after a few minutes, possibly due to the repeated desorption-reabsorption of oxygen molecules. When the UV light is turned off, the photogeneration of electron-hole pairs is stopped, and only a small fraction of unpaired electrons is reabsorbed by oxygen molecules, resulting in a current decay. Additional factors may contribute to strong current decrease under the UV illumination. For example, some charge carriers may be trapped in pores in the cellulose film.

#### 3.2.2. Exposure side effect

**Figure 7.**
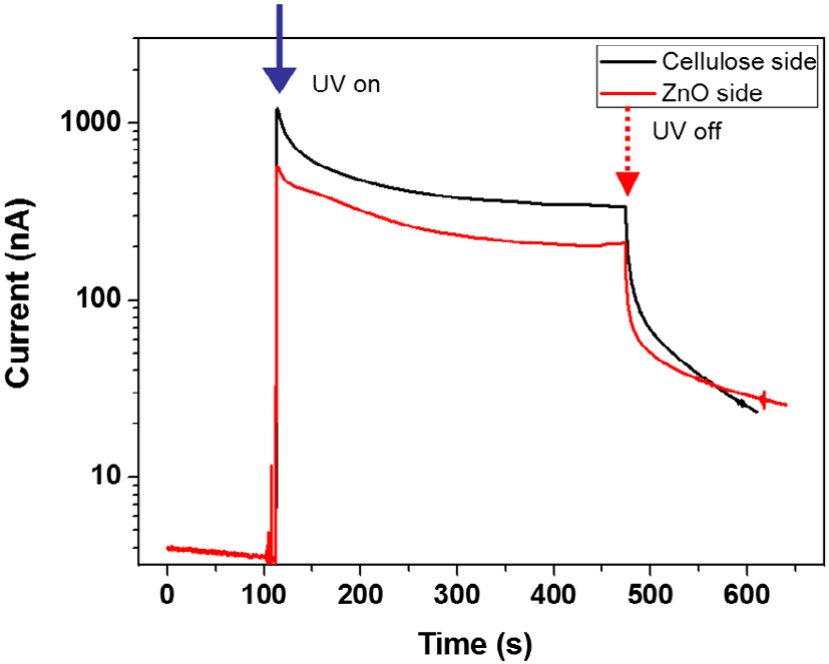
Bi-directional photoresponse behavior of the CEZOHN.

The prepared CEZOHN has two different sides: the ZnO nanorods side and the cellulose film side. To verify the effect of UV exposure sides, UV was exposed to the ZnO side and the cellulose side separately. Figure 7 shows the bi-directional photoresponse behavior of the CEZOHN. The cellulose side shows a higher induced current than the ZnO side. ZnO nanorods absorb UV light between 315 and 380 nm wavelength in UV as shown in Figure 4(b). This range of UV light is almost absorbed by the ZnO nanorods. However, since the CEZOHN has a 120 ~ 150 nm thick seeding layer, only 5 ~ 7% reduction of the transmittance peak at 370 nm was observed on the cellulose side. This phenomenon of the cellulose side resulted in higher current output than the ZnO side. This might also be associated with the low light scattering of the cellulose side. Once light is exposed to the cellulose side, it penetrates through the cellulose film and reaches the ZnO seed layer, which is much smoother than the ZnO nanorods layer, resulting in low scattering. In brief, the induced current with the cellulose side exposure is at least 20% higher than the ZnO side.

#### 3.2.3. Electrode effect

**Figure 8.**
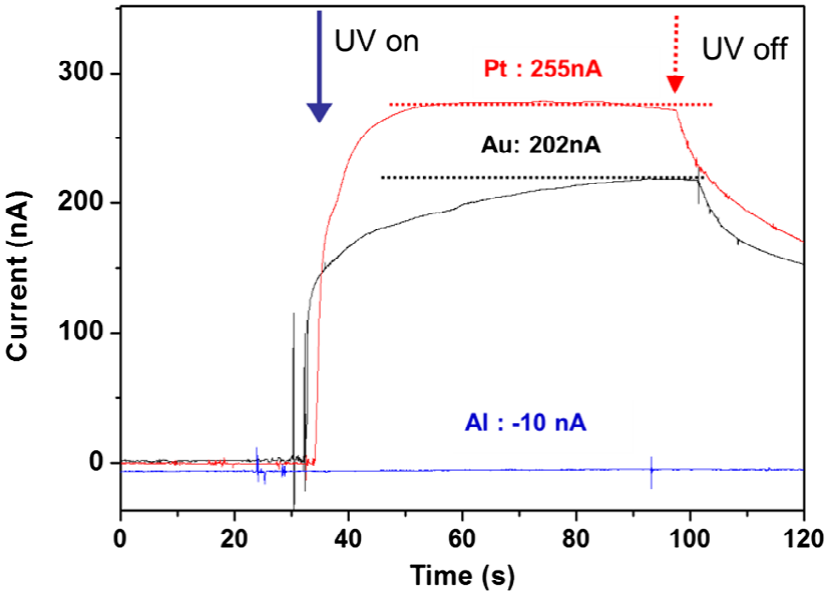
UV photocurrent for two different electrodes.

Metal layers are necessary on both sides of the CEZOHN to form electrodes. In order to study the effect of electrodes, Al, Au and Pt were deposited on both sides of the composite. Figure 8 shows the induced current with various metals under UV exposure of 0.7 mW/cm^2^ intensity. The Pt electrode exhibited a 20% higher current output than the Au case. Note that the Al electrode did not show any photoresponse under UV exposure. This might be associated with work function values of 5.7, 5.2, 4.3 and 4.2 for Pt, Au, ZnO and Al, respectively. The big gap of work function between the electrode and ZnO induces a high photocurrent under UV exposure. Since the work function of Al electrode is almost the same as that of ZnO, the Al electrode did not show any current output.

**Figure 9.**
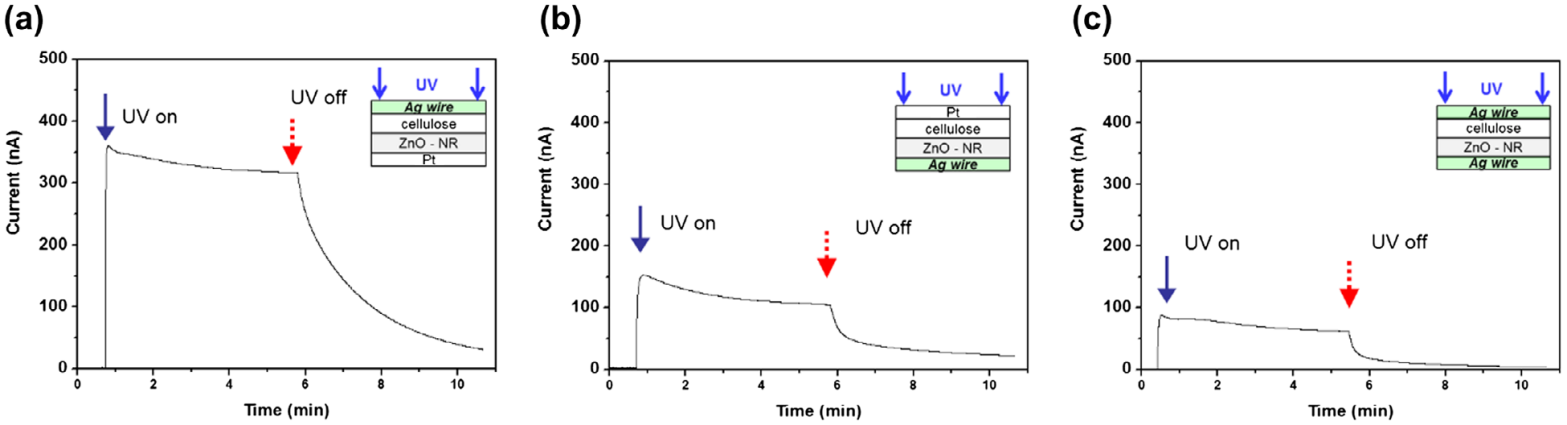
Photoresponse of three UV sensor configurations depicted in the insets.

The transparency of CEZOHN exhibits over 65% within a visible light range. It is higher than other organic-inorganic composites in which inorganic particles scatter lights so as to deteriorate its transparency. To apply transparent electronics, a transparent electrode on CEZOHN is made by depositing silver nanowires (AgNWs) using a spray. The AgNWs deposition on the cellulose film has been introduced in previous work [5]. In short, AgNWs (Nanopyxis) were dispersed in isopropyl alcohol (IPA) with 0.5 wt.% concentration. The concentration of AgNWs is controlled by diluting it with IPA. A spray gun (Paasche, H202S) with supporting external pressure control was used with 0.025 ml/cm^2^ spray volume rate on the cellulose film. The AgNWs electrode was formed in 3 different configurations. The first one is AgNWs electrode deposition on the cellulose side and the ZnO side is a Pt electrode. The second one is AgNWs for the ZnO side and Pt for the cellulose side. The third one is AgNWs electrodes on both sides. Figure 9 displays the induced current values with different electrode configurations. The first, second and third cases exhibited 300, 150 and 80 nA current output, respectively under UV exposure at 0.9 mW/cm^2^ intensity. There is a wide difference between the first and second cases, which indicates that the high photoresponse performance can be realized with Pt coating on the ZnO side. The average transmittance of the AgNWs where both sides deposited is about 52%. A transparent UV sensor can be realized with a lower concentration of AgNWs, provided the electrical conductivity of the electrode is maintained.

#### 3.2.4. Light source effect

The UV portion of sunlight may be split into three components: the UV-C range extends from 100 to 280 nm. UV-C sunlight has a relatively high intensity, but it is entirely absorbed by Earth’s ozone layer. The UV-B range lies between 280 and 320 nm. It is also largely absorbed by the atmosphere, yet prolonged exposure to UV-B light can easily ‘burn’ the human skin. UV-B light cannot penetrate through windows. The wavelength range of UV-A light extends from 320 to 400 nm. This light is not absorbed by the ozone layer; it can penetrate deeply into the human skin, affecting the immune system and potentially causing skin cancer [51,52].

**Figure 10.**
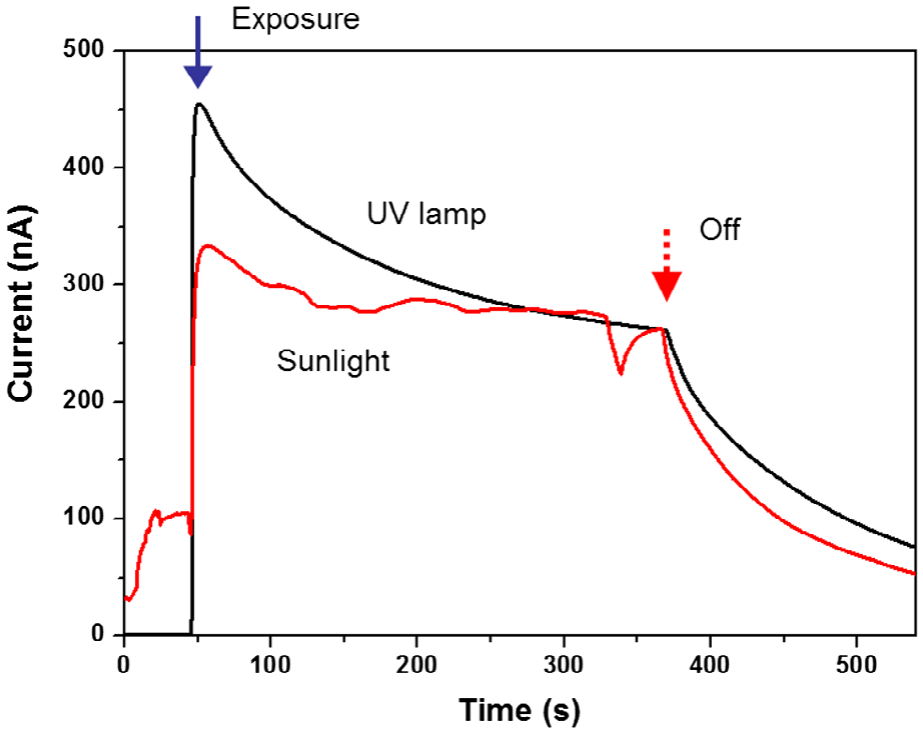
Photoresponse under sunlight or UV lamp exposure.

The prepared CEZOHN has a sharp luminescence peak at 373 nm, which means that it can detect UV-A light. Figure 10 shows a comparison of the induced currents under the UV lamp exposure and sunlight. The applied UV is sensitive at UV-A, which has the highest sensitivity at 360 nm. Measured intensities of the UV lamp and sunlight are 1.0 and 0.4 mW/cm^2^, respectively. Even with the low intensity exposure of sunlight, the photoresponse performance was similar to the UV lamp. The initially induced current under sunlight is lower than the UV lamp case, but it becomes the same after 5 minutes’ exposure. Thus, it is concluded that the CEZOHN can detect UV-A in sunlight.

#### 3.2.5. Repeat test and aging effect

**Figure 11.**
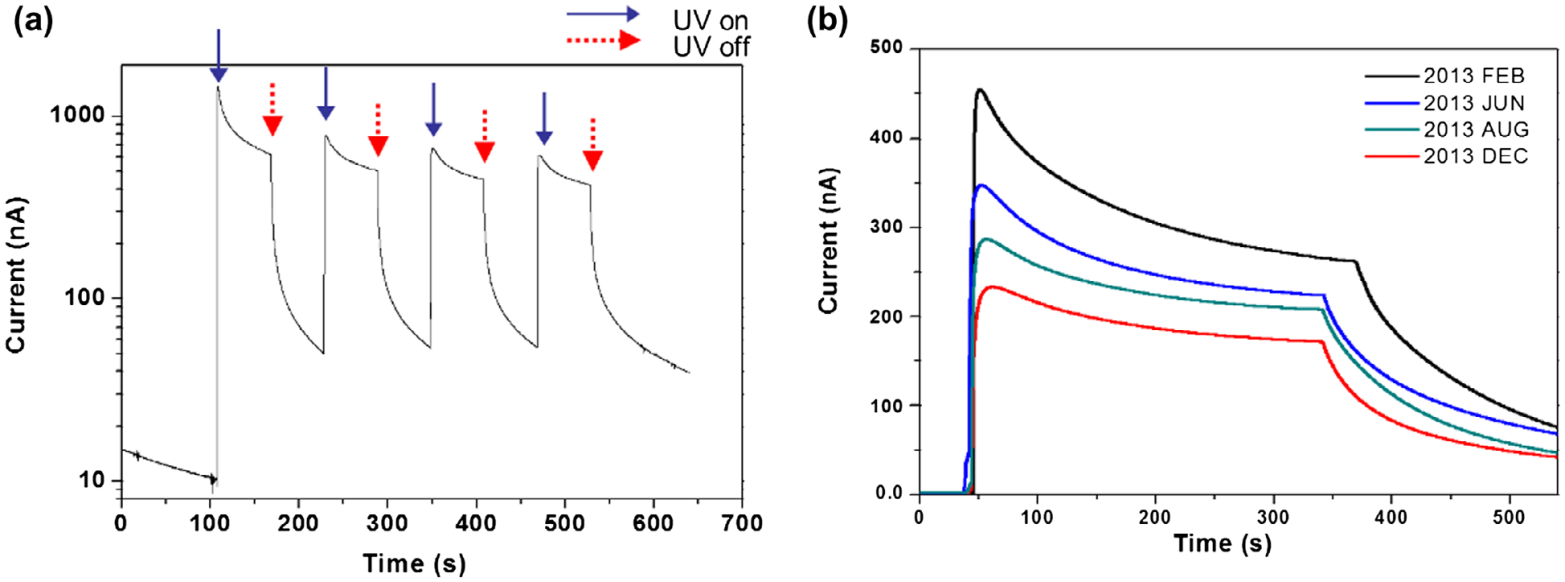
(a) Photocurrent under cyclic UV exposure and (b) long-term stability of the UV sensor.

Sensors should be fast to respond and reliable. Tests were performed repeatedly in order to investigate these characteristics. The result of a repeated test with cyclic UV exposure is shown in Figure 11(a). The cyclic exposure was composed of 1 minute UV on and 1 minute off and repeated 4 times in a cycle under 1.3 mW/cm^2^ intensity UV exposure. The CEZOHN shows a good response time of less than 1 second. The photocurrent was induced clearly with a fast response time under on and off UV exposures. The result proves that the UV sensor made with the CEZOHN has a good repeatability.

The durability of sensors is important for real applications. The aging effect is a key issue for durability. The prepared CEZOHN can be influenced by environmental moisture and temperature changes over time. Degree of usage also affects durability. The UV photoresponse of the CEZOHN was observed over a few months as shown in Figure 11(b). The induced current was 456 nA at first under 0.7 mW/cm^2^ UV intensity. The photocurrent was reduced to 76% and 62% after 4 and 6 months. After 10 months, the measured photocurrent was 234 nA, at which the performance dropped to 51%, nearly half-life of its initial performance. The UV sensor was kept in room condition without any protecting treatment. Considering this point, 10 months for half-life is quite a long time. As a further durability test, the CEZOHN was maintained even after 18 months and it was found that it maintained the half-life level of the induced current.

#### 3.2.6. Flexibility effect

**Figure 12.**
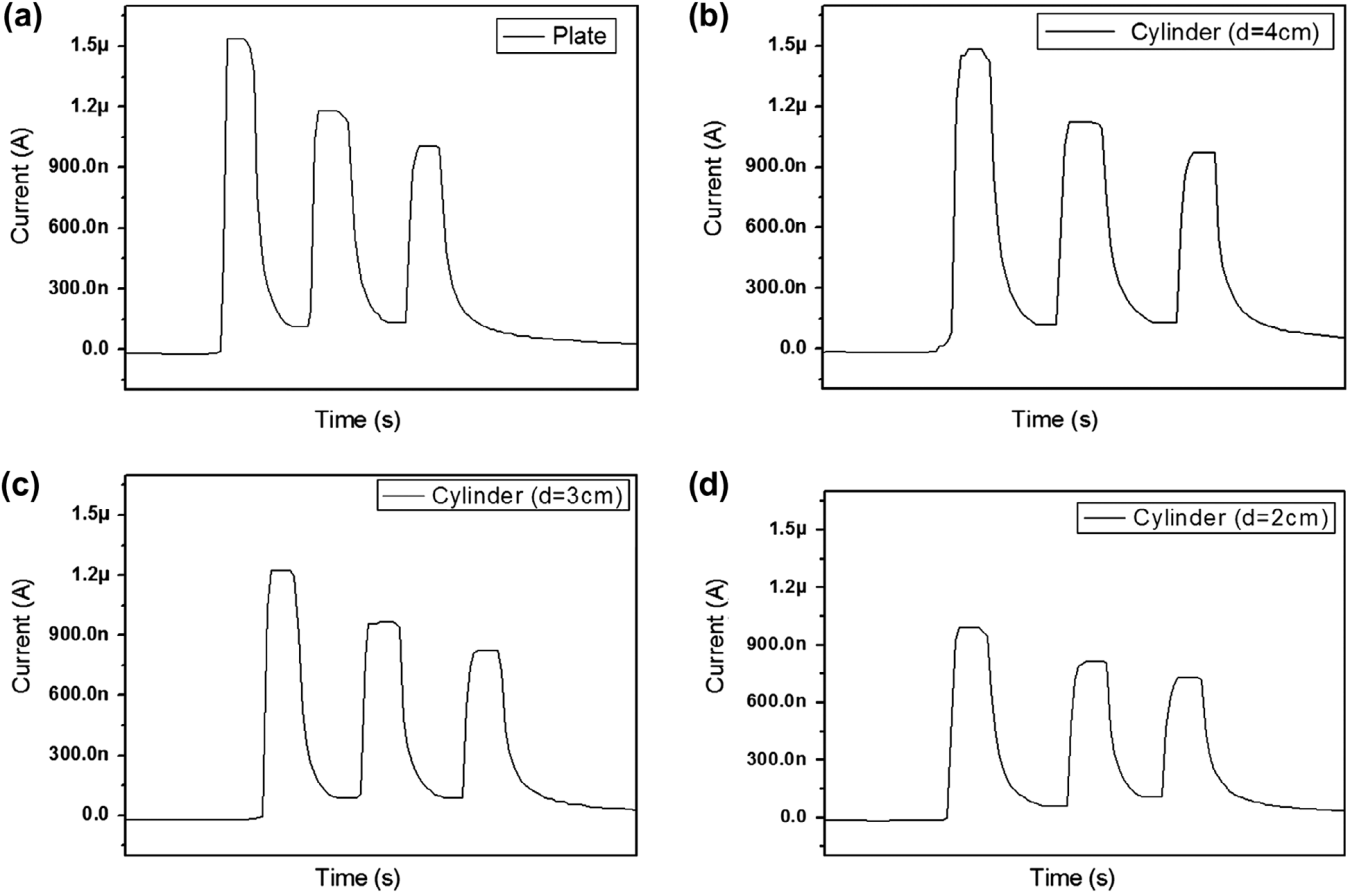
Photoresponse behavior of the UV sensor with (a) flat and (b) 4 cm, (c) 3 cm, (d) 2 cm diameter circular substrates.

Recently, flexible electronic devices have been used in wearable electronics. The CEZOHN can detect UV-A from sunlight. So, if it has certain flexibility over the sensing behavior, it may be useful for a wearable UV sensor. Thus, the flexibility test was done by attaching the UV sensor to flexible substrates with different curvatures. Several circular shape plastics were prepared for the flexible substrates. Figure 12 demonstrates the photoresponse of the UV sensor with flat and 4, 3 and 2 cm diameter circular substrates. For a reference condition, the flat substrate was used first with a fixed UV intensity of 1.5 mW/cm^2^. Figure 12(a) reveals the highest saturated induced current of 1.54 μA for the flat substrate. From the flexible substrate test, a 4 cm diameter circular substrate showed an induced current of 1.46 μA, which is similar to the flat substrate case as shown in Figure 12(b). This phenomenon is due to the size of the active exposure zone, of which the electrode size is 4 cm × 1 cm. So, the bent UV sensor on the 4 cm diameter cylinder has UV exposed to the whole active exposure zone, albeit the loss of projected zone was only due to the curvature. The induced currents are 1.22 μA and 0.98 μA for 3 and 2 cm diameter circular substrates, respectively. The induced current decreases as the diameter of the circular substrate decreases owing to an increased loss of projected zone by the UV exposure. The CEZOHN can be applied for a wearable UV sensor.

## 4. Conclusions

We have fabricated a cellulose-ZnO hybrid nanocomposite (CEZOHN) consisting of vertically aligned ZnO nanorods on the surface of cellulose film. The nanorods had the wurtzite structure and an aspect ratio of 9 ~ 11. CEZOHN was coated with electrodes for UV sensing characterization. It showed a sharp photoluminescence peak at 373 nm. The photocurrent in CEZOHN increased linearly with an intensity increase of UV light. CEZOHN exhibited bi-directional photoresponse behavior, and the cellulose side induced 20% more current than the ZnO side. The Pt electrode resulted in the highest photocurrent owing to its large work function difference with ZnO. The use of AgNWs transparent electrode with CEZOHN opened a possibility for a transparent UV sensor, which was responsive to sunlight and exhibited a short response time under repeated UV exposure. Flexibility and aging tests proved that the CEZOHN can be used for a wearable sensor that detects the UV component of sunlight with high sensitivity and stability. The sensor exploits the advantages of cellulose in terms of flexibility, low price, sustainability and transparency.

## Disclosure statement

No potential conflict of interest was reported by the authors.

## Funding

This research was supported by the Basic Science Research Program through the National Research Foundation of Korea (NRF) funded by the Ministry of Science, ICT and Future Planning [grant number NRF-2015R1A3A2066301].
